# Draft Genome Sequence of *Escherichia* Phage PGN829.1, Active against Highly Drug-Resistant Uropathogenic Escherichia coli

**DOI:** 10.1128/MRA.01141-18

**Published:** 2018-11-21

**Authors:** Naveen Chaudhary, Balvinder Mohan, Neelam Taneja

**Affiliations:** a Department of Medical Microbiology, Postgraduate Institute of Medical Education and Research, Chandigarh, India; Indiana University Bloomington

## Abstract

The Escherichia phage PGN829.1 was isolated from sewage of a tertiary care referral hospital in North India. It lyses multiple strains of highly drug-resistant uropathogenic E. coli. It belongs to the family *Podoviridae*. Its genome is closest to that of Escherichia phage Vb_EcoP_PhAPEC7.

## Abstract (precis)

The Escherichia phage PGN829.1 was isolated from sewage of a tertiary care referral hospital in North India. It lyses multiple strains of highly drug-resistant uropathogenic E. coli.

## ANNOUNCEMENT

Here, we report an annotated genome sequence of Escherichia phage PGN829.1, which is capable of lysing multiple strains of highly drug-resistant uropathogenic Escherichia coli (UPEC), which is resistant to cefotaxime, cefoperazone, amikacin, gentamicin, nitrofurantoin, nalidixic acid, ciprofloxacin, imipenem, co-trimoxazole, tazobactam-piperacillin, and cefoperazone-sulbactam. This phage was isolated from a sewage discharge of a 2,000-bed tertiary care referral center at the Postgraduate Institute of Medical Education and Research in Chandigarh, India. We used an enrichment procedure involving a spot assay and soft agar plaque assay (1). A single plaque 2 mm in diameter was picked up with a micropipette tip and transferred into 1 ml of SM buffer (10 mM MgSO_4_ · 6H_2_O) followed by vortexing to release the phages from the agar plug (2). We amplified the phage by adding 4 ml of broth culture of the host bacterial strain to 100 µl of the phage content and incubating for 24 h at 37°C. The phage was purified by ultracentrifugation with polyethylene glycol 8000 (Sigma-Aldrich, USA) and dialysis with dialysis membrane 110 (HiMedia Laboratories, India) (3). We tested the host range of PGN829.1 against multiple clinical isolates of highly drug-resistant UPEC and found it to be active against multiple UPEC strains (Table 1). We extracted the phage genomic DNA with a phage DNA isolation kit (Norgen Biotek, Canada) (4). The DNA quality was assessed using a NanoDrop 8000 spectrophotometer (Thermo Scientific, USA), and the concentration was estimated using a Qubit 3.0 fluorometer (Life Technologies, USA). An Illumina sequencing library of genomic DNA was prepared using a NEBNext Ultra library preparation kit, and sequencing was performed on an Illumina HiSeq 2500 sequencer, which generated 8,115,440 paired-end raw reads that were 100 bp long. The genome was sequenced to an average depth of 100×. *De novo* assembly was performed using Iterative Virus Assembler (IVA) version 1.0.8 (5). The FastQ files were preprocessed before performing assembly. Adapter sequences were trimmed with Cutadapt and Sickle, and we filtered out reads with an average quality score of less than 30 in any of the paired-end reads (6). We predicted genes from the IVA-assembled contigs using GLIMMER 3 software. The predicted genes were annotated using our in-house contig annotation pipeline (CANoPI) and followed a three-step procedure, (1) comparison with the UniProt database using the BLASTx program, (2) organism annotation, and (3) gene ontology (GO) annotation. In the first step, the predicted genes with an E value cutoff of 10^−3^ and identity cutoff of 40% were retained for further annotation. A total of 87 open reading frames were predicted, out of which 84 could be annotated based on significant hits in the UniProt database. The GO terms molecular function (MF), cellular component (CC), and biological process (BP) for genes were mapped using the Blast2Go tool (7). A BLASTn similarity search revealed that the genome of PGN829.1 is closest to Escherichia phage Vb_EcoP_PhAPEC7 (GenBank accession number KF562340), which belongs to a family of noncontractile short-tailed phages known as *Podoviridae* (8). Phage PGN829.1 has a genome size of 74.4 kb and a GC content of 42.9%. We conclude that the lytic activity shown by Escherichia phage PGN829.1 against highly drug-resistant UPEC strains may have potential therapeutic value for treating urinary tract infections.

**TABLE 1:** Antibiotic resistance profile of UPEC strains lysed by *Escherichia* phage PGN829.1^*a*^

Strain	CTX	CFP	GEN	AMK	NAL	NOR	CIP	SXT	NIT	IPM	TZP	CFP-SUL	PMB
*E. coli* 42829	R	R	S	S	R	R	R	R	R	S	R	R	S
*E. coli* 13879	R	R	R	R	R	R	R	R	S	R	R	R	R
*E. coli* 15286	R	R	R	R	R	R	R	R	S	R	R	R	R
*E. coli* 14075	R	R	R	R	R	R	R	R	S	R	R	R	R

a CTX, cefotaxime; CFP, cefoperazone; GEN, gentamycin; AMK, amikacin; NAL, nalidixic acid; NOR, norfloxacin; CIP, ciprofloxacin; SXT, co-trimoxazole; NIT, nitrofurantoin; IPM, imipenem; TZP, tazobactam-piperacillin; CFP-SUL, cefoperazone-sulbactam; PMB, polymyxin B; R, resistant; S, susceptible.

This study was approved by the Institute Ethical Clearance Committee of PGIMER, Chandigarh.

### Data availability.

The raw sequence reads have been submitted to the NCBI SRA under accession number PRJNA495477, and the draft genome sequence of Escherichia coli phage PGN829.1 has been deposited in NCBI GenBank under accession number MH733496.
